# Pulmonary cavernous hemangioma combined with smooth muscle hyperplasia: a case report and review of the literature

**DOI:** 10.1186/s44215-023-00048-z

**Published:** 2023-05-17

**Authors:** Takashi Ibe, Takayuki Kosaka, Masayuki Sugano, Satoru Kakizaki, Ken Shirabe

**Affiliations:** 1 Department of Thoracic Surgery, National Hospital Organization Takasaki General Medical Center, 36 Takamatsu-cho, Takasaki, Gunma 370-0829 Japan; 2 Department of General Surgical Science, Gunma University Graduate School of Medicine, Maebashi, Gunma Japan; 3 Department of Thoracic Surgery, Isesaki Municipal Hospital, Isesaki, Gunma Japan; 4 Department of Clinical Research, National Hospital Organization Takasaki General Medical Center, Takasaki, Gunma Japan

**Keywords:** Pulmonary cavernous hemangioma, Benign lung tumor, Smooth muscle hyperplasia

## Abstract

**Background:**

Cavernous hemangiomas are usually located in the liver, skin, and subcutaneous tissues. Although they can arise from any organ, cavernous hemangiomas rarely occur as a pulmonary tumor. We herein report a rare case of a pulmonary cavernous hemangioma that was surgically resected.

**Case presentation:**

A woman in her 40s was found to have 2 well-defined nodules in the lower lobe of the left lung by computed tomography during following up of bladder cancer. She had a history of surgery for tetralogy of Fallot at 6 years old and pulmonary valve replacement for pulmonary valve insufficiency in her late 30s. She had also undergone surgery for bladder cancer. Although there was no accumulation of ^18^F-fluorodeoxyglucose on positron emission tomography (PET), the tumor slowly grows. Surgical resection was therefore performed to obtain a definitive diagnosis. The postoperative histological examination revealed an encapsulated nodule comprising large, dilated vessels lined with vascular endothelium and filled with blood, which led to the diagnosis of a pulmonary cavernous hemangioma.

**Conclusion:**

We experienced a rare case of pulmonary cavernous hemangioma and reviewed the previous reports.

## Background

Cavernous hemangiomas are benign diseases composed of large, dilated vascular spaces lined by a single layer of endothelial cells and filled with blood [1, 2]. They are usually located in the liver, skin, and subcutaneous tissues [1]. Although they can arise from any organ, cavernous hemangiomas rarely occur as a pulmonary tumor [3–5]. A previous review of pulmonary cavernous hemangiomas (PCHs) found only 10 previously reported cases during the 60 years prior to 2010 [6]. Zhuang et al. also summarized 17 cases of PCH reported in the database of PubMed, Embase, and Web of Science from 1996 to 2017 [7].

While most PCHs are clinically benign, they can be difficult to differentiate from metastases when occurring in multiples in patients with malignancy [2]. Although cough and hemoptysis are the most common symptoms, asymptomatic patients have been reported. Furthermore, PCHs rarely but occasionally cause sudden death due to blood aspiration [8].

We experienced a rare case of PCHs during the follow-up of bladder cancer that was surgically resected. We further reviewed previous reports and summarize 43 total cases of PCH [2–31], including our own, making this review the largest ever with regard to the number of patients involved.

## Case presentation

A woman in her 40s consulted our department because of pulmonary nodules. She had been found to have lung nodules by computed tomography (CT) performed for a routine follow-up of bladder cancer. She had a history of surgery for tetralogy of Fallot at 6 years old and pulmonary valve replacement for pulmonary valve insufficiency in her late 30s. She had also undergone surgery for bladder cancer. She had no specific family history. She had been prescribed aspirin due to her history of surgery for pulmonary valve insufficiency.

**Fig. 1:**
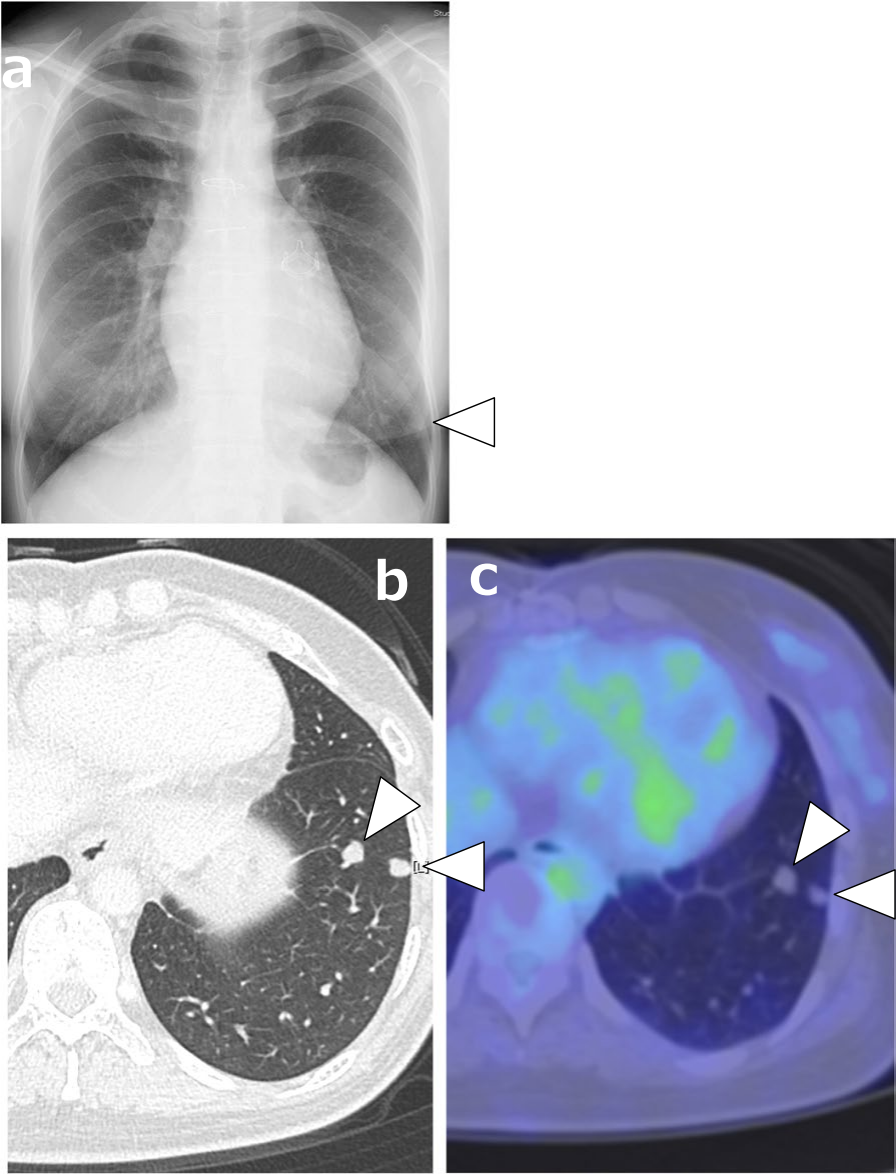
**a** Chest X-ray findings: small nodular shadow (arrowhead) was observed in the left lower lung field. **b** Chest computed tomography findings: two well-demarcated solid nodules (arrowheads) were observed in the segment 8 region of the left lower lobe, 9 mm and 7 mm in diameter. **c**^18^F-fluorodeoxyglucose positron emission tomography (FDG-PET) findings: no accumulation was observed in pulmonary nodules (arrowheads) or other areas

Chest X-ray showed a small nodular shadow in the left lower lung field (Fig. 1a). CT in the lung window setting showed abnormal nodules measuring 8 and 6 mm in the lower lobe of the left lung of segment 8 (Fig. 1b). These nodules were difficult to visualize under the mediastinal window setting. As a result, contrast-enhanced CT in the mediastinal window setting did not depict the pulmonary nodules nor had an enhanced effect. There was no accumulation of ^18^F-fluorodeoxyglucose (FDG) on positron emission tomography (PET) of the tumor (Fig. 1c). Because the nodules were small, were existing in a unilateral localized area, and showed no accumulation of FDG, they seemed benign. The attending physician therefore decided to follow them conservatively. However, after 3 years of follow-up, the nodules showed a slow growth. The patient was thus referred to our department for surgical resection to obtain a definitive diagnosis with differentiation from nontuberculous mycobacteria, metastatic lung tumor, and lung cancer.

Her clinical status was unremarkable, and she was thought to have enough surgical endurance. She had a surgical wound after median sternotomy and a systolic murmur at the left sternal border of the second to third intercostals. Laboratory examinations showed no remarkable changes, except for a slight elevation of brain natriuretic peptide (BNP) levels at 142 pg/mL. Tumor markers, including carcinoembryonic antigen (CEA), cytokeratin fragment (CYFRA) 21, and pro-gastrin-releasing peptide (Pro-GRP), were within the normal ranges. *Cryptococcus neoformans* antigen, aspergillus antigen, serum (1,3)-beat-d-glucan, and the T cell spot (T-SPOT) test were also negative. Soluble interleukin-2 receptor (sIL-2R) showed no elevation.

The multiple pulmonary nodules showed a slow growth and she had a surgical history of bladder cancer. We could not rule out that the metastases originated from bladder cancer. For that reason, we considered a biopsy of the pulmonary nodules, rather than follow them conservatively. But the nodules were small in diameter, existing in the peripheral of the left lower lobe. Therefore, a non-surgical biopsy such as transbronchial lung biopsy (TBLB) or CT-guided lung biopsy was thought to be difficult to make a definite diagnosis. With the preoperative diagnosis of a metastatic tumor originating from bladder cancer, we planned a surgical lung biopsy.

We performed wedge resection of the left lower lung, including the nodules, via three-port video-assisted thoracic surgery (VATS). During the operation, we confirmed a pulmonary nodule just below the pleura in S8 of the left lower lobe by palpation. In addition, we could also directly palpate the other deeper lesion inside the lung parenchyma.

After grasping the two nodules with ring-like grasping forceps to secure a sufficient surgical margin from the lesions, we resected the two pulmonary nodules in bulk with an autosuture device. After resection, we confirmed the sufficient margin from the deeper lesion by checking the cutting surface of the specimen.

Intraoperative histological examination showed the nodule consisted of spindle-shaped cells with oval nuclei and eosinophilic cytoplasm, forming cystic cavities abundantly. Intraoperative histological diagnosis was suspicious of lymphangiomyomatosis, obtained by examining one of the two nodules. The surgery time was 68 min, and the bleeding volume was small.

**Fig. 2:**
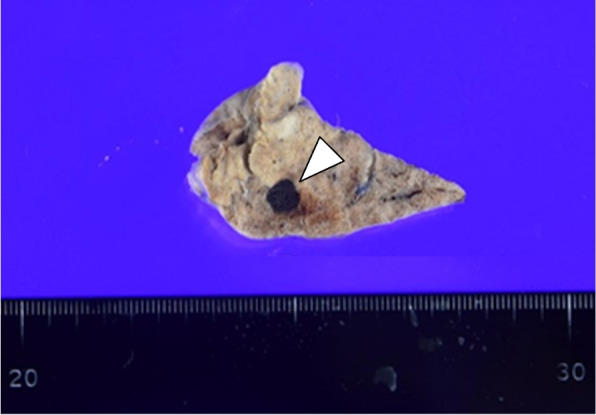
Macroscopic findings of the resected specimen. The cut surface of one of two resected nodules. The nodule used for intraoperative histological diagnosis is not presented because it was not stored. A well-demarcated nodular lesion (arrowhead) was found in the peripheral region of the resected specimen at the left lower lobe

**Fig. 3:**
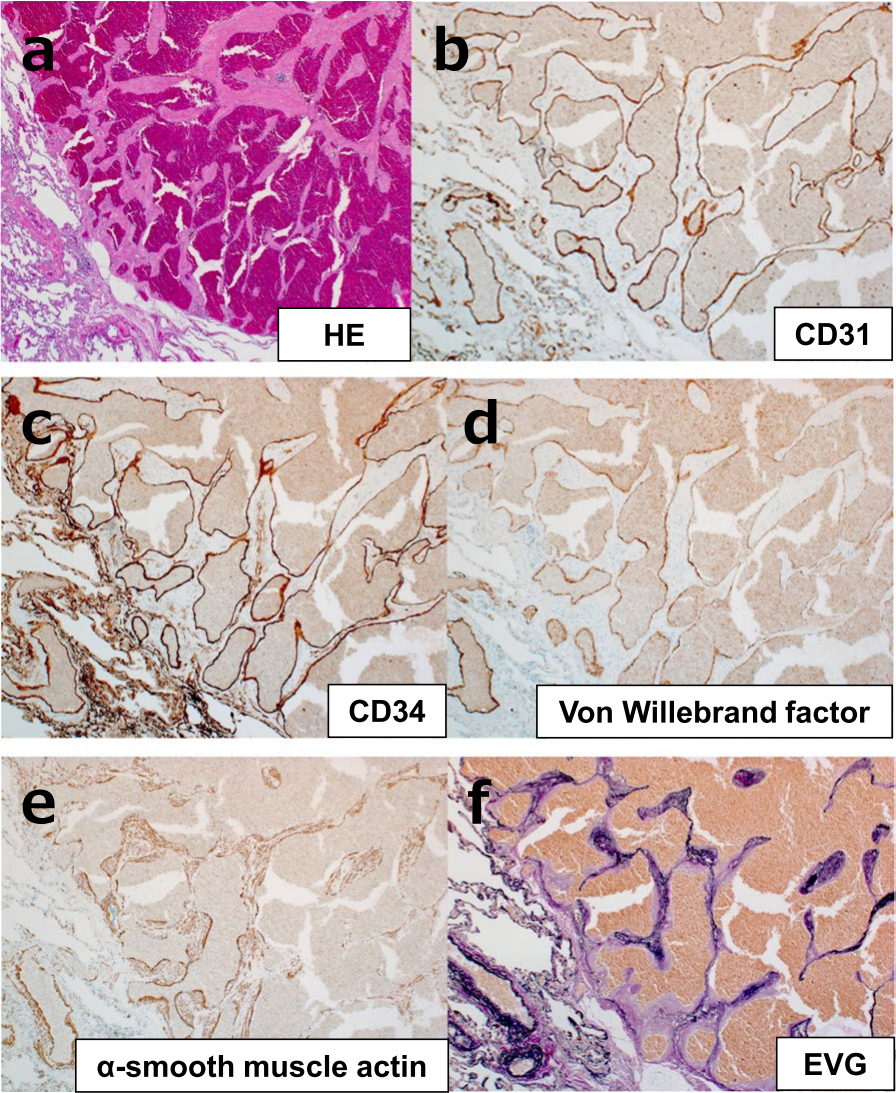
Microscopic and immunohistochemical findings of the resected specimen. **a** Hematoxylin-eosin staining (low power field). The inside of a well-demarcated nodule consisted of the dilated vascular lumen with vascular endothelial cells and smooth muscle components. The vascular endothelial cells were histological findings of cavernous hemangioma, and no malignant features were observed. Although the smooth muscle proliferated, it did not surround the lumen of the vessel and was considered unlikely to be neoplastic. **b**–**f** Immunohistochemical staining (low power field) showed CD31- (**b**), CD34- (**c**), and von Willebrand factor (vVF)-positive endothelial cells (**d**) in the vascular lumen and α-smooth muscle actin-positive cells in the smooth muscle component (**e**). Elastic van Gieson (EVG) staining (**f**) showed irregular elastic fibers, and the distribution of elastic fibers was continuous in the surrounding lung tissue and hemangioma tissue, indicating that the smooth muscle cells did not form a neoplastic vessel wall. Based on the above findings, the lesion was considered to be pulmonary cavernous hemangioma complicated by smooth muscle hyperplasia

Gross findings of the resected specimen showed a well-demarcated nodule in the S8 peripheral part of the left lung (Fig. 2). Gross finding of the nodule used for intraoperative histological diagnosis is not presented because it was not stored. The resected two nodules had the same findings of histological examination. A histological examination revealed an encapsulated nodule comprising large, dilated vessels lined with endothelium and filled with blood (Fig. 3).

The nodule consisted of vascular endothelial and smooth muscle components. Immunohistochemistry showed the endothelial cells lining the inner lumen to be positive for anti-CD31, anti-CD34, and anti-von Willebrand factor (vWF) antibody, which are immunohistochemical markers of vessels. The smooth muscle component was positive for α-smooth muscle actin. Irregular elastic fibers were observed by elastic van Gieson (EVG) staining, and the distribution of elastic fibers was continuous in the surrounding lung tissue and hemangioma tissue, indicating that smooth muscle cells did not form a neoplastic vascular wall.

We finally diagnosed the patient with PCH with smooth muscle hyperplasia. The postoperative course was uneventful, and the patient was discharged after the surgery. Currently, a CT follow-up is underway at the outpatient department.

## Discussion

Hemangiomas are benign vascular tumors commonly seen in the head and neck region [1, 2]. They are usually classified as capillary hemangioma, cavernous hemangioma, large-vessel hemangioma, skeletal muscle hemangioma, intravascular papillary endothelial hyperplasia, spindle cell hemangioma, and hobnail hemangioma based on their clinical appearance and the caliber of the vessel involved [1]. Cavernous hemangioma, a relatively uncommon variant, is histologically composed of tangles of capillaries or widely dilated vascular channels [1, 2]. Although cavernous hemangioma can occur throughout the body, it rarely occurs as a pulmonary tumor [2–5].

Ishikawa et al. summarized the 11 cases of PCH in 2010 [6]. Zhuang et al. also summarized 17 cases of PCH reported in the database of PubMed, Embase, and Web of Science from 1996 to 2017 [7], including their own case. We summarized these previous reports, including these two review reports, and searched the databases again, including PubMed, Embase, and Web of Science. We finally collected a total of 43 patients, including our present case (Table 1) [2–31]. The age of the affected patients ranged from 10 weeks to 84 years old, with the highest incidence occurring in the 5th decade of life. Hemorrhaging was seen independent of age. Among the 43 patients with PCHs, 21 were men and 22 were women. Twenty of the 43 patients were asymptomatic, including our case. Among the symptomatic cases related to PCH, hemoptysis (*n*=8), cough (*n*=6), and hemosputum (*n*=4) were the most common symptoms. Life-threatening massive hemoptysis was reported in only one case [8]. The symptoms were not related to the size of the hemangioma [2–31]. The number of pulmonary lesions in the reported cases was solitary in 27 of 43 cases and multiple in 16 of 43 cases.

**Table 1:** Summary of previous case reports of PCH

Cases	Year	Author	Age (years)	Sex	Symptoms	Number of PCHs	Treatment or outcome
1	1948	Cleland WP [9]	51	F	Cough, sputum, heart failure	Single	Lobectomy
2	1950	Forsee JH [10]	20	M	Asymptomatic	Single	Lobectomy
3	1954	Sano K [11]	43	F	Hemoptysis	Single	Lobectomy
4	1957	Oohane Y [12]	51	F	Hemosputum	Single	Lobectomy
5	1958	Goodall JF [13]	66	M	Cough, hemoptysis	Single	(Autopsy)
6	1963	Tsunekawa K [11]	58	F	Asymptomatic	Single	Lobectomy
7	1966	Noda E [12]	24	F	Loss of weight	Single	Lobectomy
8	1976	Ichikawa K [11]	65	F	Hemosputum	Single	Enucleation
9	1977	Katsumura T [11]	65	F	Hemosputum	Single	Enucleation
10	1983	Ikeda M [11]	67	M	Cough	Single	Enucleation
11	1985	Mori A [11]	61	F	Asymptomatic	Multiple	Autopsy
12	1989	Tanaka H [11]	7	M	Asymptomatic	Single	Excision
13	1990	Sugiyama S [11]	58	F	Hemosputum	Single	Lobectomy
14	1992	Kawamata O [11]	22	M	Asymptomatic	Single	Lobectomy
15	1992	Galliani C [14]	10 weeks	M	Rhinorrhea, cough, low-grade fever	Multiple	Lobectomy
16	1993	Karino T [11]	55	F	Asymptomatic	Single	Excision
17	1996	Wu JM [3]	7	M	Chest pain, hemoptysis, dyspnea	Multiple	Interferon alfa-2a
18	1996	Ienaga H [15]	45	M	Asymptomatic	Single	Thoracoscopic surgery
19	1998	Nakamura A [11]	46	M	Asymptomatic	Single	Lobectomy
20	1999	Taniguchi T [12]	36	F	Asymptomatic	Single	Partial resection
21	2000	Kase M [16]	29	F	Asymptomatic	Single	Thoracoscopic surgery
22	2003	Fujita A [17]	44	F	Asymptomatic	Single	Partial resection
23	2003	Kobayashi A [5]	15	F	Asymptomatic	Multiple	No remarkable progression under observation for more than 2 years
24	2003	Sirmali M [8]	54	M	Hemoptysis	Single	Lobectomy
25	2004	Fine SW [2]	84	M	Repeated falls unrelated to PCHs	Multiple	Dead due to cardiovascular disease
26	2006	Maeda R [4]	54	M	Asymptomatic	Single	Thoracotomy
27	2008	Giannopoulos S [18]	38	M	Fever, dyspnea, hemoptysis	Multiple	No data
28	2009	Basile U [19]	73	M	Hemoptysis, dyspnea	Single	Lobectomy
29	2010	Ishikawa T [6]	73	F	Cough, spitting blood	Single	Dead due to massive blood aspiration
30	2010	Kunitani K [20]	16	M	Asymptomatic	Single	Partial resection
31	2011	Lovrenski A [21]	67	M	Pneumonia	Single	Thoracotomy
32	2014	Matsubara S [22]	29	F	Vaginal hemorrhage unrelated to PCHs	Multiple	Follow-up
33	2014	Chen QR [23]	19	F	Dyspnea and fatigue	Multiple	Thoracoscopic biopsy and follow-up with no apparent symptoms
34	2014	Yang LL [24]	36	M	Pleural effusion	Multiple	Dead because of heart and respiratory failure 2 months later
35	2014	Jia CY [25]	54	F	Intermittent cough and sputum	Single	Thoracotomy
36	2015	Miyamoto U [26]	61	M	Asymptomatic	Multiple	Follow-up
37	2017	Wang C [27]	35	F	Chest pain	Multiple	Liver transplant for hepatic hemangioma and surgically removed for cardiac hemangioma
38	2018	Zhuang BW [7]	78	M	Dizziness unrelated to PCHs	Multiple	Follow-up
39	2019	Gandhi S [28]	24	M	Hemoptysis	Single	Lobectomy
40	2020	Ancel J [29]	35	F	Hemoptysis	Multiple	Lobectomy
41	2020	Bae K [30]	72	F	Asymptomatic	Multiple	Video-assisted thoracoscopic biopsy and follow-up
42	2021	Lee JH [31]	71	M	Asymptomatic	Multiple	Wedge resection for diagnosis
43	2022	Our case	40	F	Asymptomatic	Multiple	Wedge resection by video-assisted thoracic surgery

*PCH* Pulmonary cavernous hemangioma, *[ ]* reference number

The preoperative diagnosis is difficult because the clinical symptoms and imaging findings are scarce, and the imaging findings are similar to those of other malignant and benign pulmonary lesions. Radiologically, PCHs show solitary or multiple nodular lesions with no characteristic features. Fine and Whitney stated that calcification in PCH was indicative of organized thrombosis in the veins [2]. In the present case, chest CT did not show calcification of the nodules. The preoperative diagnosis was difficult in all previously reported cases as well, and a diagnosis was only made during or after surgery. Bae et al. [30] proposed the usefulness of dual-layer spectral CT for the diagnosis of PCH. Contrast-enhanced CT is useful for the diagnosis of liver cavernous hemangioma [32]. It may be also useful for the diagnosis of PCH above a certain size. In our case, the nodules were small and it was difficult to visualize under the mediastinal window setting.

In many cases, resection was performed for diagnostic and therapeutic purposes including enucleation, wedge resection, and lobectomy depending on the location and size of the hemangioma [2–31]. In the case of solitary lung lesions, if a definitive diagnosis can be obtained during surgery, wedge resection or enucleation that preserves the lung function is desirable, as it is a benign lesion. However, for multiple lung lesions, complete resection of all lesions is not realistic. A careful follow-up is a good option for asymptomatic small lesions. No recurrence after complete resection has been reported thus far.

None of the previous reports pointed out a link to hemangiomas based on the presence of smooth muscle hyperplasia. In our case, smooth muscle cells were not neoplastic and did not surround the vascular lumen. The relationship between cavernous hemangioma and smooth muscle hyperplasia is unclear. Further analyses and the accumulation of cases will be needed to clarify this relationship.

## Conclusion

We reported a rare case of PCHs and reviewed previous reports of similar cases. Cavernous hemangiomas are rarely seen in the lung. A preoperative diagnosis of PCH is difficult, and surgical resection is recommended as the treatment of choice for solitary lesions. If a tumor is diagnosed as PCH during the operation, wedge resection or enucleation is favored because of the benign behavior of such lesions.

## Data Availability

The datasets used and/or analyzed during the current study are available from the corresponding author on reasonable request.

## Abbreviations

PCH: Pulmonary cavernous hemangiomas
CT: Computed tomography
FDG-PET: 18F-fluorodeoxyglucose on positron emission tomography
BNP: Brain natriuretic peptide
CEA: Carcinoembryonic antigen
CYFRA 21: Cytokeratin fragment 21
Pro-GRP: Pro-gastrin-releasing peptide
T-SPOT: T cell spot
sIL-2R: Soluble interleukin-2 receptor
TBLB: Transbronchial lung biopsy
VATS: Video-assisted thoracic surgery
vWF: von Willebrand factor
EVG: Elastic van Gieson
